# The construction and analysis of tumor-infiltrating immune cells and ceRNA networks in metastatic adrenal cortical carcinoma

**DOI:** 10.1042/BSR20200049

**Published:** 2020-03-27

**Authors:** Runzhi Huang, Ziqi Liu, Tingli Tian, Dianwen Song, Penghui Yan, Huabin Yin, Peng Hu, Xiaolong Zhu, Yihan Liu, Zhenyu Li, Tong Meng, Jie Zhang, Zongqiang Huang

**Affiliations:** 1 Department of Orthopaedics, The First Affiliated Hospital of Zhengzhou University, Zhengzhou 450052, China; 2 Division of Spine, Department of Orthopedics, Tongji Hospital affiliated to Tongji University School of Medicine, Shanghai 200065, China; 3 Tongji University School of Medicine, Tongji University, Shanghai 200092, China; 4 School of Mathematical Sciences of Tongji University, Shanghai 200092, China; 5 Department of Orthopedics, Shanghai General Hospital, School of Medicine, Shanghai Jiaotong University, Shanghai 200080, China; 6 Shanghai East Hospital, Key Laboratory of Arrhythmias, Ministry of Education, Tongji University School of Medicine, Shanghai 200120, China

**Keywords:** Adrenal cortical carcinoma, ceRNA network, CIBERSORT, Immune cell, Metastasis, Nomogram

## Abstract

**Purpose:** To construct and analyze tumor-infiltrating immune cell and ceRNA (competitive endogenous RNA) networks in metastatic adrenal cortical carcinoma (ACC).

**Methods:** A ceRNA network was established to identify the ceRNAs involved in metastasis of ACC based on 92 samples from TCGA, including 18 cases of metastasis and 74 cases of non-metastatic primary tumors. And the algorithm “cell type identification by estimating relative subsets of RNA transcripts (CIBERSORT)” was used to quantify the proportion of immune cells in ACC. In addition, predictive nomograms based on the types of important immune cells or ceRNAs were constructed to predict ACC prognosis. Moreover, we evaluated the relationships between metastatic ACC-specific immune cells and ceRNA networks to identify the potential immune gene characteristics.

**Results:** Ten prognostic biomarkers were identified as key members of the ceRNA network and three tumor-infiltrating immune cells were identified by CIBERSORT algorithm. Some important co-expression patterns between immune cells and ceRNAs network indicate significant correlation between Macrophages M0 and hsa-miR-130b-3p (*P* < 0.001), Macrophages M0 and H2AFX (*P* = 0.003).

**Conclusions:** The present study inferred that the metastasis-related ceRNAs of H2AFX, hsa-miR-130b-3p and Macrophages M0 might play important roles in ACC metastasis.

## Introduction

Adrenal cortical carcinoma (ACC) is a malignant neoplasm originating from the adrenal cortex with an annual incidence of (0.7–2.0)/1 million people [1]. The prognosis for ACC is poor with an overall 5-year survival of <40% [2]. ACC can be classified into functional and nonfunctional adrenal cortical carcinoma according to corticosteroid secretion [3]. ACC is highly malignant and aggressive with a poor prognosis [4]. It usually has metastasis at the time of diagnosis. Patients with metastatic ACC has poorer prognosis and the total survival time is less than 1 year [5]. Therefore, it is necessary to explore the potential mechanisms of ACC metastasis.

Molecular and cellular biomarkers in pathological diagnosis play an important role in predicting metastasis and prognosis of ACC [3,5]. Among them, metastasis-related ceRNAs and tumor-infiltrating immune cells have aroused our interest. However, few previous studies have paid attention to them. Non-coding genes can usually regulate the expression of genes to a certain extent. At the transcriptional level, lncRNA regulated the expression of both mRNA and microRNA by changing chromatin modification and mRNA stabilization [6]. MicroRNAs post-transcriptionally regulated gene expression [7]. A competing endogenous RNA (ceRNA) network is a transcriptional regulatory network at the molecular level, composed of lncRNAs, miRNAs (miRNAs) and mRNAs, among which microRNA response element (MRE) is the core element of the network [8]. In ceRNA network, the one-to-many and many-to-one regulatory relationships among transcription factors, miRNAs and target genes may take part in gene regulation, thus affecting the biological characteristics of tumors [8]. At the cellular level, the assessment of the extent and type of tumor infiltrating immune cells have been proved to be of great significance in predicting metastasis and mortality [9,10]. However, there is no joint network to predict the metastasis of ACC. Therefore, it is necessary to do better research on tumor infiltrating immune cells and ceRNA networks.

In the present study, we screened genes significantly related to survival from ACC-related data in TCGA database, and established a ceRNA network based on gene expression profiles. In the meantime, “cell type identification by estimating relative subsets of RNA transcripts (CIBERSORT)” was used to quantify the proportion of immune cells in ACC [11]. Based on the ceRNA network and CBERSORT analysis, two nomograms were constructed to predict the prognosis of ACC. Moreover, we evaluated the relationships between metastatic ACC-specific immune cells and ceRNA networks to identify potential immune gene characteristics.

## Materials and methods

### Data collection and differential gene expression analysis

In the present study, in order to obtain the differential gene between recurrent and *in situ* adrenal cortical carcinoma, we downloaded the RNA profiles of adrenal cortical carcinoma and metastasis samples from the TCGA (https://portal.gdc.cancer.gov/projects/TCGA-ACC) database. Among them, 92 cases were selected, including 18 cases of metastasis and 74 cases of non-metastatic tumors.

The edgeR method was used to analyze the differentially expressed genes in metastatic and non-metastatic tumors. When false discovery rate (FDR) *P* value < 0.05, log2(Fold Change) > 1.0 and log2(Fold Change) < -1.0 were defined as up-regulated and down-regulated genes, respectively.

### Construction of the ceRNA network

Before primary statistical analysis, the experimental validation-based information on the miRNA–mRNA interaction was downloaded from miRTarBase (http://mirtarbase.mbc.nctu.edu.tw/) [12], and the lncRNA–microRNA interaction information was downloaded from lncbase v.2 Experimental Module (http://carolina.imis.athena-innovation.gr/diana_tools/web/index.php?r=lncbasev2%2Findex-experimental) [13]. The databases are based on experimental validation. Then, based on the above data, using Cytoscape v.3.5.1, we calculated the maximal information coefficient (MIC) of lncRNA, miRNA and RNA, and selected miRNAs, lncRNAs and mRNAs which showed significant results in hypergeometric detection and correlation analysis to construct ceRNA network [14].

### Survival analysis and nomograms of key members in the ceRNA network

Kaplan–Meier survival analysis showed the relationship between the expression of biomarkers and the prognostic value shown in ceRNA network and the survival outcomes of ACC patients. Subsequently, by screening the significant variables in the initial Cox model, the important biomarkers were included in the Cox proportional hazard model to illustrate the variables with prognostic values. All the important biomarkers were integrated into Cox model, and lasso regression was used to judge whether the model was over fitted. Cox regression model is a kind of linear regression using shrinkage, in which the data value shrinks to a specific point to ensure the applicability of multiple models. Finally, we established a multivariate model-based nomogram to predict the prognosis of ACC patients. According to the expression level of biomarkers with prognostic value, we can get the points of each biomarker and add them together to get the total points, thus showing the total survival probability of 3 and 5 years. At the same time, calibration curves and receiver operating characteristic curves (ROC) were used to evaluate the resolution and accuracy of the nomogram.

### CIBERSORT estimation

To further investigate the cytological causes of bone metastasis in adrenocortical carcinoma and the molecular mechanism of important biomarkers in ceRNA network to some extent, CIBERSORT (http://cibersort.stanford.edu/) algorithm was used to estimate the proportion of 22 immune cell types in ACC (patients with or without metastasis) [11]. Samples with CIBERSORT output value *P* < 0.05 are considered eligible for further analysis. The Wilcoxon rank-sum test was used to search for immune cells. In addition, Cox regression and Kaplan–Meier methods were also used to evaluate the relationship between the proportion of immune cells and the overall survival of ACC patients. The proportion of the prognostic markers and metastasis related immune cells was scored to determine the relationship between the most relevant immune cells and ceRNA for ACC recurrence.

Only bilateral *P* < 0.05 was considered statistically significant. Institute of Statistics and Mathematics, Vienna, Austria (Package: GDCRNATools [15], edgeR, ggplot2, RMS, planet, preprocessCore, curvilinear, timeROC).

### Multidimensional validation

To reduce the error, several databases including Cell marker [16], LncRNA2Target [17], Metascape [18], Ontogene [19], String [20], Oncomine [21], Cancer Cell Line Encyclopedia (CCLE), cBioPortal for Cancer Genomics [22], genotype-tissue Expression (GTEx) [23], Gene Expression Profiling Interactive Analysis (GEPIA) [24], LinkedOmics [25], SurvExpress [26] and The Human Protein Atlas (Proteomics. Tissue-based map of the human proteome) detected the gene and protein expression levels of key biomarkers at the tissue and cell levels. OncomiR [27] was used to explore the correlation between clinical features and key miRNAs.

## Results

### Identification of significantly differentially expressed genes

**Figure 1:**
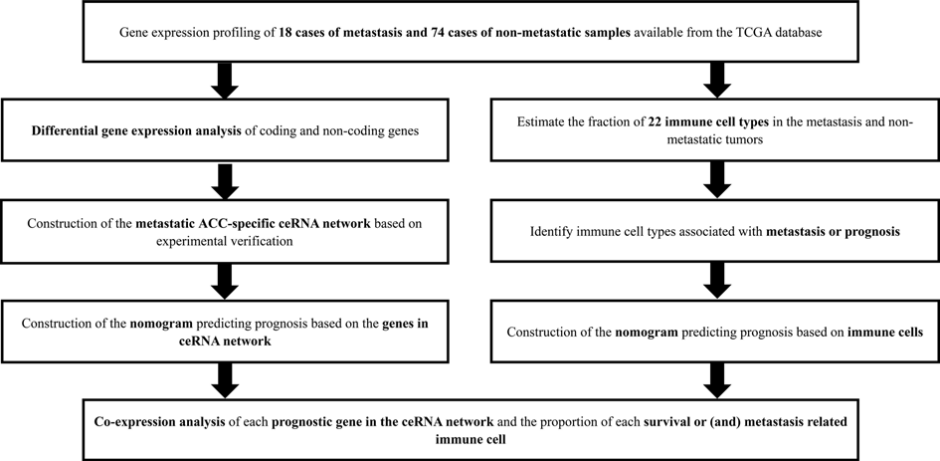
The flow chart of the analysis process Based on TCGA database, 92 ACC cases were included, and the ceRNA network was constructed by multi database analysis. Immune cells associated with ACC recurrence and survival were identified by CIBERSORT estimation. The co expression of important immune cells and important genes in ceRNA was analyzed to determine the relationship between immune cells and ceRNA that are most related to ACC recurrence.

Figure 1 illustratess the analysis process of the present study. The demographic information of the samples from the TCGA is summarized in Supplementary Table S1. Most of the samples were white and female, with an average age of 47.16 years.

**Figure 2:**
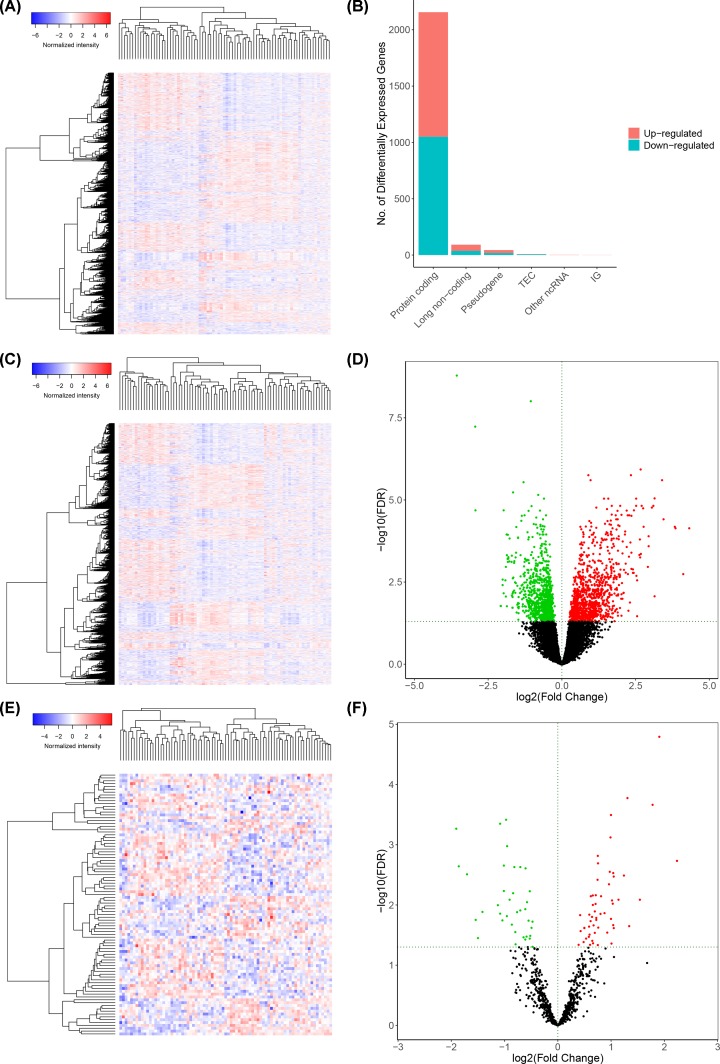
The results of differential expression gene analysis The heatmap (**A**) and type plot (**B**) of all differentially expressed genes, heatmap (**C**) and volcano plot (**D**) of differentially expressed mRNA and heatmap (**E**) and volcano plot (**F**) of differentially expressed lncRNA in metastatic and non-metastatic adrenal cortical carcinomas. In volcano plot, the red dot represents the up-regulated gene and the green dot represents the down regulated gene. Genes with the log (fold-change) > 1.0 or < -1.0 and FDR < 0.05 were defined as the differential expression genes.

In the 60483 RNAs in the TCGA database, we identified 92 differentially expressed lncRNAs (37 down-regulated and 55 up-regulated), 2156 differentially expressed mRNAs (1051 down-regulated and 1105 up-regulated) which red represent up-regulated, green represent down-regulated (Figure 2).

### Construction of the ceRNA network and survival analysis

**Figure 3:**
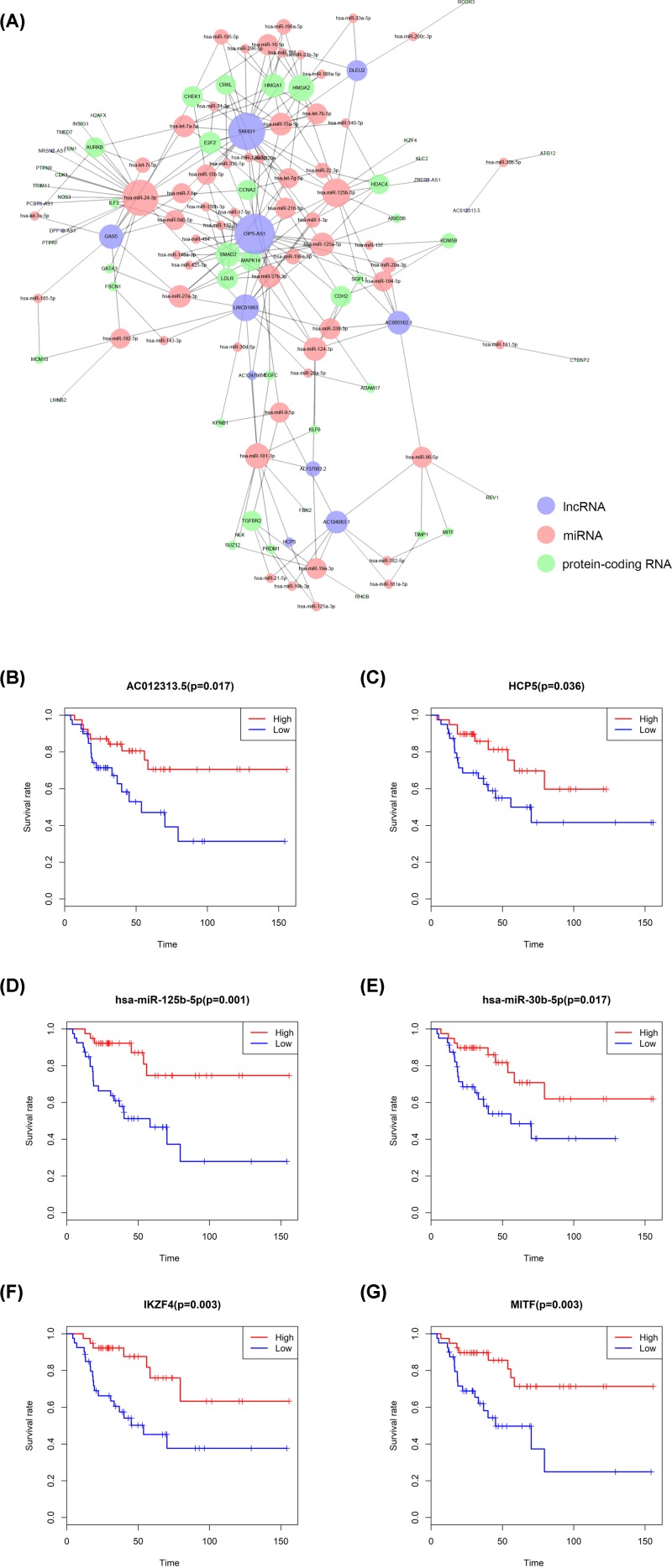
Construction of metastasis-specific ceRNA network The ceRNA network related to metastasis of adrenal cortical carcinoma, in which blue circles represent lncRNAs, red circles represent miRNAs and green circles represent protein-coding RNAs (**A**). Kaplan–Meier survival curves analysis of AC012313.5 (Novel Transcript), HCP5, has-miR-125b-5p, has-miR-30b-5p, IKZF4, MITF of the ceRNA network (**B**–**G**).

**Figure 4:**
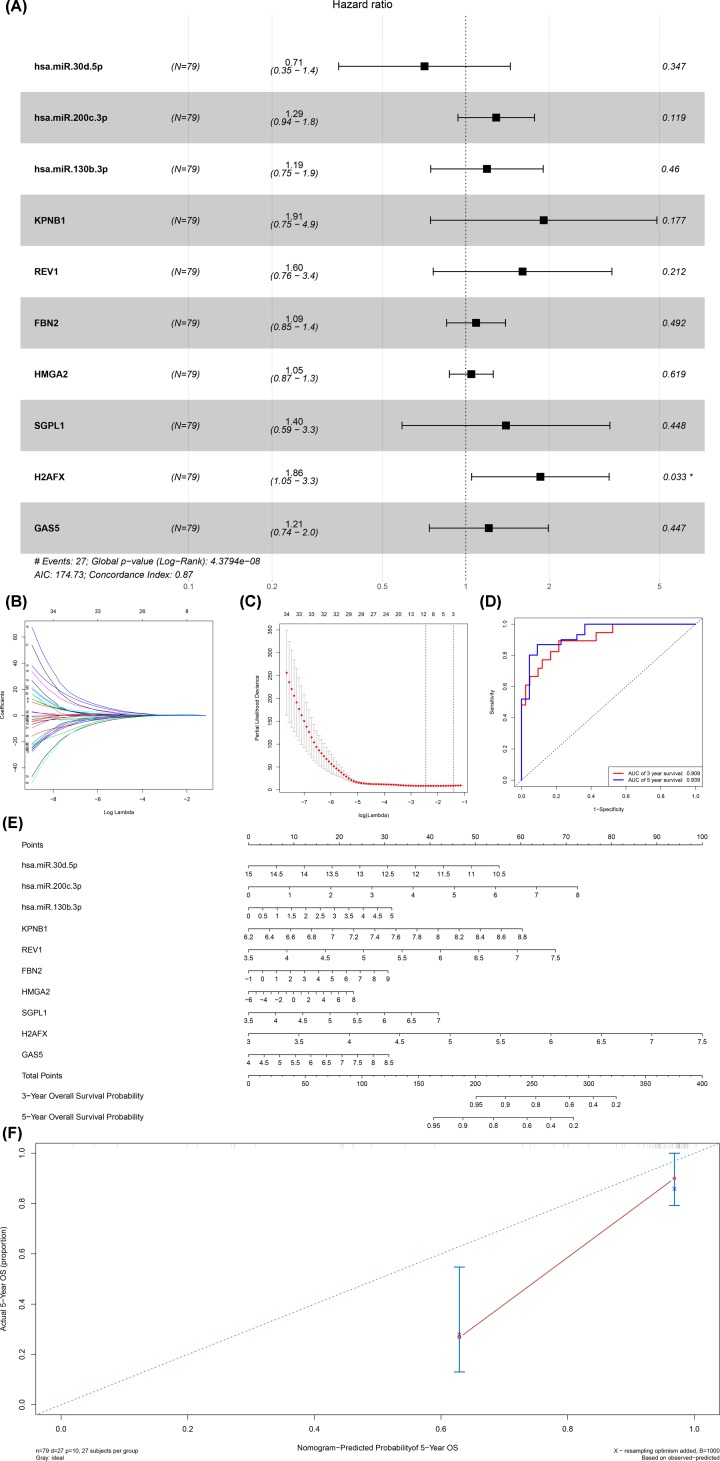
Construction and model diagnosis of prognostic nomogram including key members of ceRNA network The results of the multivariate Cox regression (**A**), nomogram (**E**) and model diagnosis process (**B,C,D** and **F**) based on the key members in the ceRNA network. hsa-miR-30d-5p, hsa-miR-200c-3p, hsa-miR-130b-3p, KPNB1, REV1, FBN2, HMGA2, SGPL1, H2AFX and GAS5 were incorporated into the Cox proportional hazards model. Nomograms for predicting patients’ prognosis were constructed based on the Cox model (**E**). Lasso regression results show that there is no over fitting (**B** and** C**). Receiver Operating Curve (ROC) (**D**) and calibration curve were used for assessing the accuracy and discrimination of the nomogram (**F**). Area Under Curve (AUC) of the 3- and 5-year survival was 0.909 and 0.939, respectively.

The ceRNA network was composed of 15 lncRNAs, 59 miRNAs and 65 mRNAs (Figure 3A). Cox regression and Kaplan–Meier method were used to study the relationship between biomarkers and metastasis in the ceRNA network. Table 1 shows the results of hypergeometric testing and correlation analysis ceRNAs interaction relationship with absolute value of correlation coefficient more than 0.50. Kaplan–Meier analysis showed that 34 RNAs were significantly correlated with the metastasis of ACC. We selected AC01231313.5 (*P* = 0.017), HCP5 (*P* = 0.036), hsa-miR-125b-5p (*P* = 0.001), hsa-miR-30b-5p (*P* = 0.017), IKZF4 (*P* = 0.003), IKZF4 (*P* = 0.003), MITF (*P* = 0.003), and displayed the KM Survival Curve of these RNAs in Figure 3B–G). Ten prognostic biomarkers were identified as key members of the ceRNA network and integrated into a new multivariate model (Figure 4A). H2AFX was a statistically significant risk factor (*R* = 1.86, 95% CI: 1.05–3.3). The regression model was visualized by the nomogram (Figure 4E). Lasso regression results showed that all ten genes were necessary for modeling (Figure 4B,C). In addition, the ROC curve showed that the 3-year survival rate (AUC) was 0.909, the 5-year survival rate (AUC) was 0.939 (Figure 4D), and the COX regression chart showed that the accuracy of the prediction was acceptable (Figure 4F). The enrichment analysis of DEGs in the ceRNA network were also performed, which revealed significant enrichment of immune system and genetic material biological processes or pathways (Supplementary Figure S1).

**Table 1:** Hypergeometric testing and correlation analysis results of ceRNAs interaction relationship with absolute value of correlation coefficient more than 0.50

LncRNA	PcRNA	MiRNAs	Correlation *P*	Hypergeometric *P*	Cor
DPP10-AS1	FSCN1	hsa-miR-24-3p	7.25559E-11	0.016769838	0.644594923
SNHG1	ILF3	hsa-miR-590-5p,hsa-miR-7-5p	1.26705E-09	0.019128962	0.609338025
OIP5-AS1	SMAD2	hsa-let-7g-5p,hsa-miR-125a-5p, hsa-miR-132-3p,hsa-miR-148a-3p, hsa-miR-15b-5p,hsa-miR-27a-3p, hsa-miR-27b-3p,hsa-miR-425-5p, hsa-miR-484	7.12396E-08	0.02621027	0.551021941
SNHG1	HDAC4	hsa-miR-125a-5p,hsa-miR-1-3p, hsa-miR-140-5p,hsa-miR-22-3p	2.59259E-07	0.03814998	0.529666277
OIP5-AS1	CRKL	hsa-miR-126-3p,hsa-miR-15a-5p, hsa-miR-320a,hsa-miR-335-5p	4.33955E-07	0.032879261	0.520723002
DPP10-AS1	PTPRF	hsa-miR-24-3p	4.49362E-07	0.001872659	0.520107919

Abbreviations: ceRNA, competing endogenous RNA; LncRNA, long non-coding RNA; MiRNA, microRNA; PcRNA; protein-coding RNA.

### Composition of immune cells in ACC

**Figure 5:**
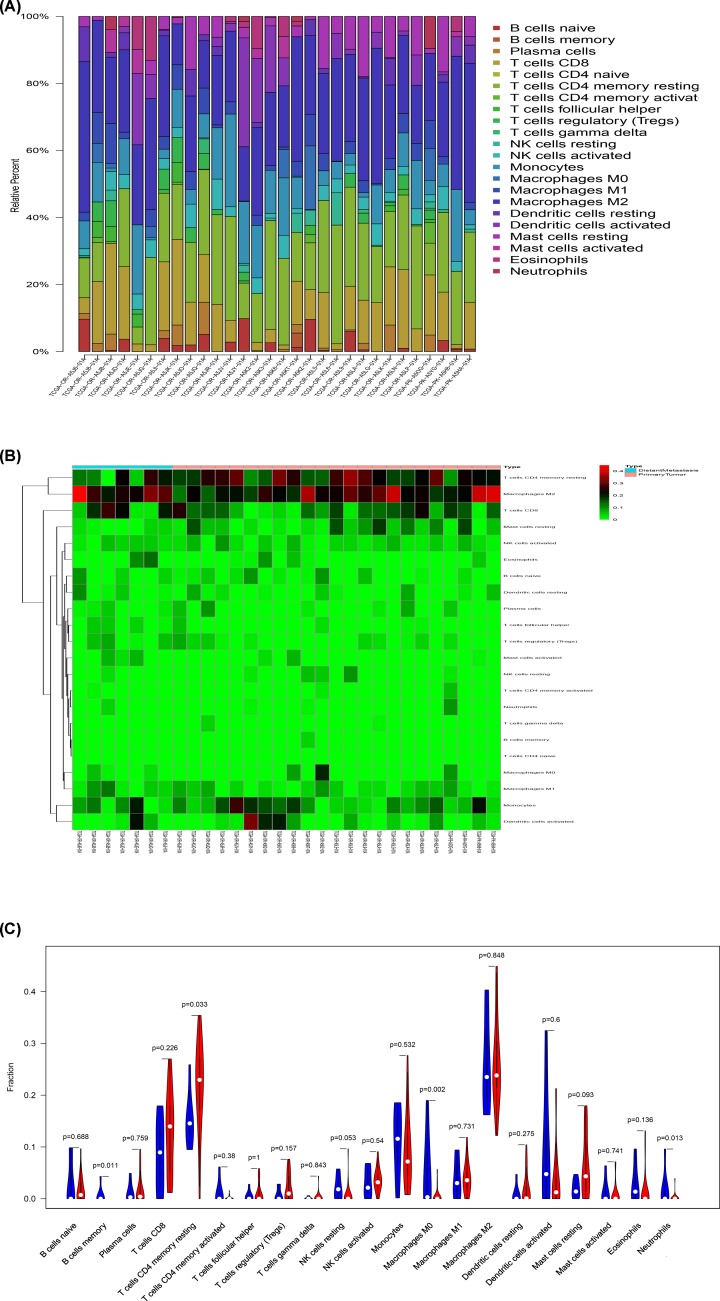
The results of CIBERSORT analysis The composition of immune cells in adrenal cortical carcinoma estimated by CIBERSORT algorithm (**A** and **B**), and the recognition of immune cells significantly associated with tumor metastasis (**C**). Bar plot showing cell types and relative percent in adrenal cortical carcinoma tissues. Different colors represent different cell types, which are listed in the right as *y*-axis, while *x*-axis represents different samples (A). Heatmap of tumor-infiltrating cells in tumor tissues in patients with the metastatic and non-metastatic adrenal cortical carcinomas. Annotations on top show clustering of samples. While the blue represents the metastatic adrenal cortical carcinomas, the red symbolizes the primary ones (B). Violin plot for comparing cells’ proportion between the metastatic and non-metastatic adrenal cortical carcinomas. It showed that B cells memory (*P* = 0.011), T cells CD4 memory resting (*P* = 0.033), neutrophils (*P* = 0.013) and macrophages M0 (*P* = 0.002) were significantly different between metastatic and non-metastatic tumors (C).

ACC immune cells estimated by CIBERSORT algorithm are displayed in histogram (Figure 5A). The metastatic and non-metastatic ACC immune cells estimated by CIBERSORT algorithm are shown in the thermogram (Figure 5B). In addition, the Wilcoxon rank-sum test showed that B cells memory (*P* = 0.011), T cells CD4 memory resting (*P* = 0.033), neutrophils (*P* = 0.013) and macrophages M0 (*P* = 0.002) were significantly different between metastatic and non-metastatic tumors (Figure 5C).

### Integrated analysis of immune cells, genes and prognosis

**Figure 6:**
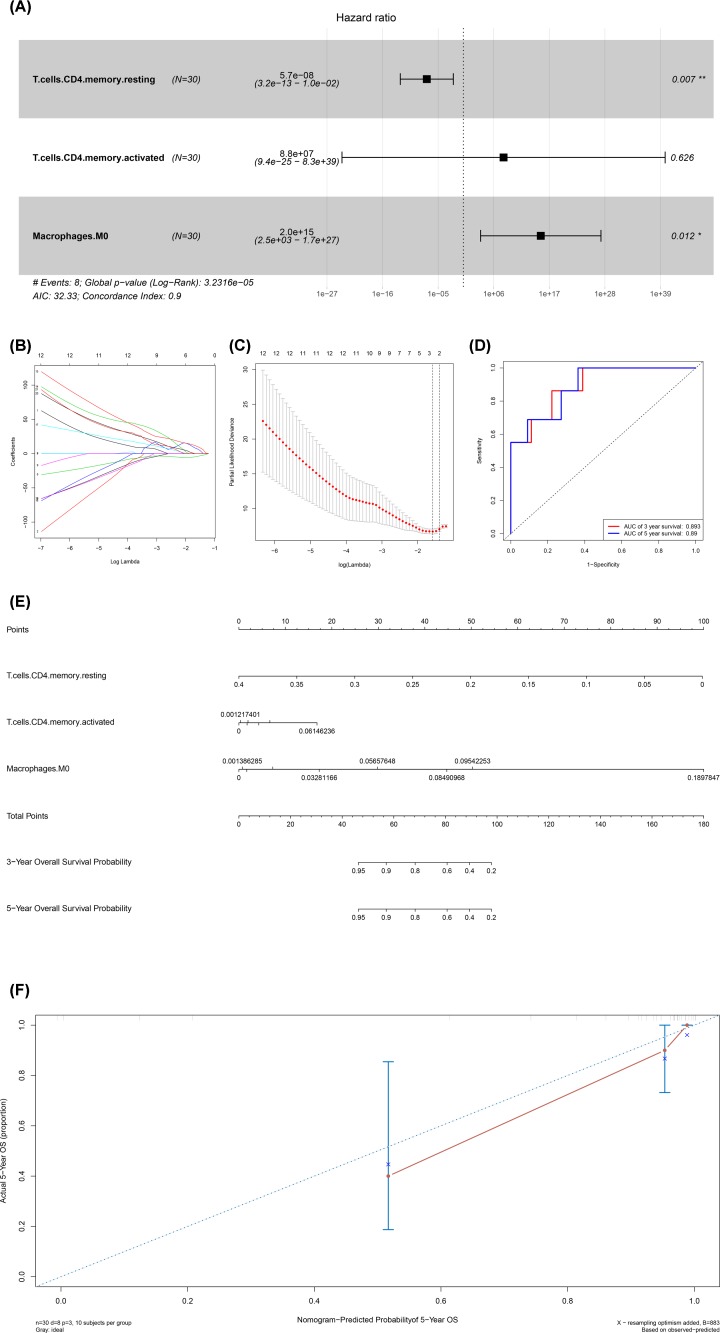
Construction and model diagnosis of prognostic nomogram including metastasis related immune cells The results of the multivariate Cox regression (**A**), Kaplan–Meier survival curve (**E**), nomogram (**F**) and model diagnosis process (**B**–**D**) based on metastasis related immune cells. B cells memory (*P* = 0.011), T cells CD4 memory retaining (*P* = 0.007), macrophages M0 (*P* = 0.012) and neutrophils (*P* = 0.013) were considered as independent predictors of the prognosis of ACC patients in the final Cox model (**A**–**D**). The nomogram based on the multivariate model was constructed (**F**). Results of the Lasso regression showed that the model was not over-fitting (**B** and **C**). The calibration curve and Receiver Operating Curve (ROC) showed good predictability (Area Under Curve (AUC) of 3-year survival: 0.893; AUC of 5-year survival: 0.890).

**Figure 7:**
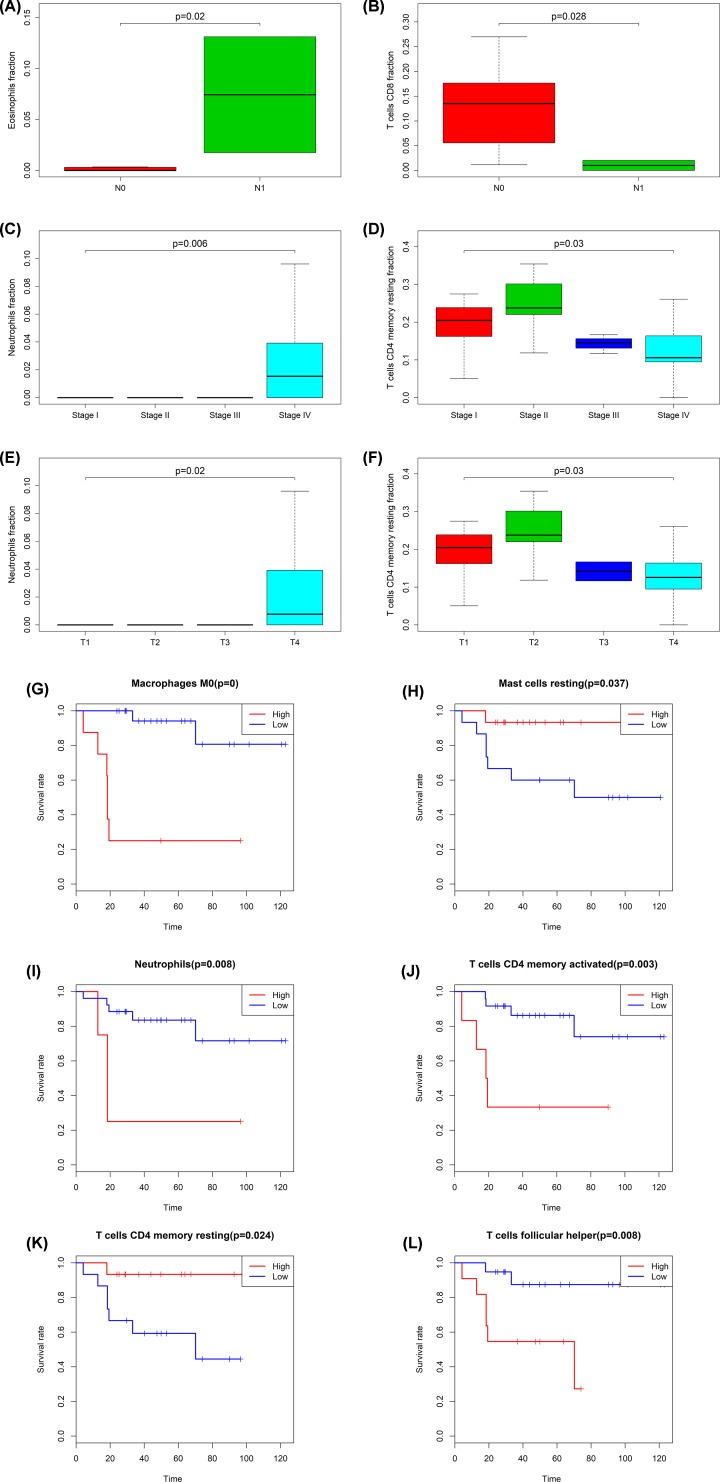
The results of clinical correlation analysis of immune cells in ACC The boxplots of clinical correlation analysis of immune cells in adrenal cortical carcinoma (**A**–**F**) and Kaplan–Meier survival curves of immune cells significantly associated with survival (**G**–**L**). Eosinophils fraction (*P* = 0.02) (**A**) and T cells CD8 fraction (*P* = 0.028) (**B**) were associated with n-stage. Clinical stage related immune cells: neutrophils fraction (*P* = 0.006) (**C**), T cells CD4 memory rest fraction (*P* = 0.03) (**D**), neutrophils fraction (*P* = 0.02) (**E**), T cells CD4 memory rest fraction (*P* = 0.03) (**F**). Macrophages (*P* < 0.001) (**G**), mast cells resting (*P* = 0.037) (**H**), neutrophils (*P* = 0.008) (**I**), T cells CD4 memory activated (*P* = 0.003) (**J**), T cells CD4 memory resting (*P* = 0.024) (**K**) and T cells follicular helper (*P* = 0.008) (**L**) were significantly correlated with survival.

**Figure 8:**
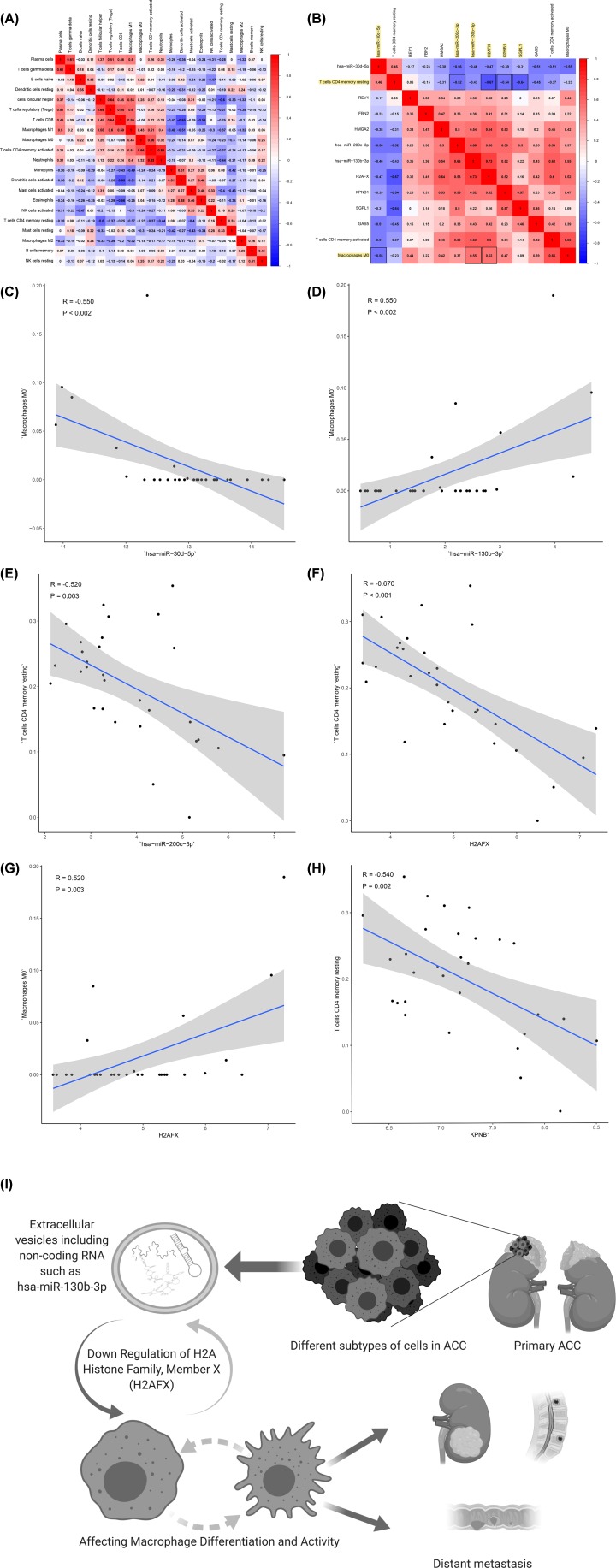
The results of co-expression analysis and the speculative mechanism diagram including metastasis-specific ceRNAs and immune cells with co-expression patterns The co-expression patterns among fractions of three immune cells and ten key members in the ceRNA network (**B**) and the correlation analysis of the proportion of immune cells (**A**). Linear relationship between immune cells and genes with high col-linearity (*P* > 0.5 or *P* < -0.5) (**C**–**H**). The results are as follows: T cells CD4 memory resting and hsa-miR-200c-3p (*R* = -0.520, *P* = 0.003), T cells CD4 memory resting and H2AFX (*P* = -0.670, *P* < 0.001), T cells CD4 memory resting and KPNB1 (*R* = -0.540, *P* = 0.002), T cells CD4 memory resting and SGPL1 (*R* = -0.640, *P* < 0.001), macrophages M0 and hsa-miR-30d-5p (*R* = -0.550, *P* < 0.002), macrophages M0 and hsa-miR-130b-3p (*R* = 0.550, *P* < 0.002), macrophages M0 and H2AFX (*R* = 0.520, *P* = 0.003). A speculative mechanism diagram of the core scientific hypothesis (**I**).

All immune cells were integrated into an initial Cox regression model. After the Lasso regression screening, B cells memory (*P* = 0.011), T cells CD4 memory retaining (*P* = 0.007), macrophages M0 (*P* = 0.012) and neutrophils (*P* = 0.013) were considered as independent predictors of the metastasis of ACC patients in the final Cox model (Figure 6A–D). Similarly, we constructed the nomogram based on the multivariate model (Figure 6F). Results of cable regression showed that the model was not over-fitting (Figure 6B,C). In addition, the calibration curve and ROC showed good predictability (AUC of 3-year survival: 0.893; AUC of 5-year survival: 0.890). Kaplan–Meier survival analysis showed a significant correlation between risk level defined by the multivariable model and survival (*P* < 0.001) (Supplementary Figure S2). Figure 7 shows the boxplots of clinical correlation analysis of immune cells in adrenal cortical carcinoma (A-F) and Kaplan–Meier survival curves of immune cells significantly associated with survival (G-L). We performed co-expression analysis of immune cells and biomarkers significantly associated with overall survival. Figure 8 illustrates important co-expression patterns between three immune cells and ten key members of the ceRNA network as well as a speculative mechanism diagram, indicating a significant correlation between T cells CD4 memory resting and hsa-miR-200c-3p (*R* = -0.520, *P* = 0.003), T cells CD4 memory resting and H2AFX (*P* = -0.670, *P* < 0.001), T cells CD4 memory resting and KPNB1 (*R* = -0.540, *P* = 0.002), T cells CD4 memory resting and SGPL1 (*R* = -0.640, *P* < 0.001), Macrophages M0 and hsa-miR-30d-5p (*R* = -0.550, *P* < 0.002), Macrophages M0 and hsa-miR-130b-3p (*R* = 0.550, *P* < 0.002), Macrophages M0 and H2AFX (*R* = 0.520, *P* = 0.003).

### Multidimensional validation

We used multiple databases for external validation to explore the gene and protein expression of differentiated genes KPNB1, H2AFX, SGPL1 and key cellular markers of T cells and macrophages in ACC, normal adrenal tissue and cell lines. First, cell markers of T cells CD4 memory were identified by Cell Marker database as CCR7, CD27 (Gene symbol: BTLA), CXCR5, IL7R, and cell markers of macrophages M0 as CD68, CD163, CD14, CD11b (Gene symbol: ITGAM) and CD206 (Gene symbol: MRC1). Through String database, the protein–protein interaction networks of differentiated gene KPNB1, H2AFX, SGPL1 and corresponding cell marker were displayed (Supplementary Figure S3). In Oncomine database, KPNB1 (*P* < 0.001) was significantly differentially expressed between ACC and normal adrenal tissue, H2AFX (median rank 3.0, COPA = 11.927) and SGPL1 (median rank 2301.0, COPA = 4.230) were abnormal up-regulated in ACC in the outlier analysis across multiple studies (Supplementary Figure S4). Besides, comprehensive analysis of genomics and clinical data in cBioPortal database showed that CCR7, CXCR5, IL7R CD68, CD163, CD14, ITGAM, MRC1 and H2AFX had genomic alteration in primary ACC (Supplementary Figure S5A). In addition, H2AFX had significant co-expression patterns with markers of T cells CD4 memory CCR7 (*P* = 0.046), IL7R (*P* < 0.001) and macrophage M0 markers CD163 (*P* = 0.037), CD14 (*P* = 0.020), ITGAM (*P* = 0.043) and MRC1 (*P* = 0.038) in ACC (Supplementary Figure S5B–G). KPNB1 also had significant co-expression with CCR7 (*P* = 0.010), ITGAM (*P* = 0.016) and MRC1 (*P* = 0.021) (Supplementary Figure S5H–J). The differentially expressed genes H2AFX and KPNB1 were co-expressed in ACC (*P* = 0.030). Furthermore, in the normal adrenal tissues in the GTEx database, KPNB1, H2AFX, SGPL1 had significant co-expression patterns with almost all cell markers (CCR7, IL7R CD68, CD163, CD14, ITGAM, MRC1) (Supplementary Figure S6). Besides, GEPIA database analysis also showed the co-expression pattern of H2AFX and CXCR5 in ACC and normal adrenal tissues (Supplementary Figure S7). The LinkedOmics database showed that KPNB1, H2AFX, SGPL1 had significant co-expression of many immune-related proteins in ACC (Supplementary Figure S8). Additionally, the analysis results of SurvExpress suggested that these genes have significant predictive value for prognosis (Censoring event: overall death, Hazard Ratio = 8.36 (95% CI, 2.88–24.31), *P* < 0.001) (Supplementary Figure S9). The Human Protein Atlas data mining results showed that proteins of H2AFX, SGPL1, CCR7, BTLA, IL7R, CD68, CD163, ITGAM could not be detected in normal tissues, and KPNB1 had low expression in normal adrenal tissues (Supplementary Figure S10). Finally, the analysis results of OncomiR database suggested that hsa-mir-30d-5p and hsa-mir-200c-3p were significantly associated with metastasis in a variety of tumors (Supplementary Table S2).

## Discussion

In the present study, we first found significant differences in the expression of tumor-infiltrating immune cells and RNAs between metastatic and non-metastatic ACCs. A ceRNA network consisting of 65 mRNAs, 15 lncRNAs and 59 microRNAs was constructed, and tumor-infiltrating immune cells were analyzed by CIBERSORT and the co-expression of key members of the important tumor-infiltrating immune cells and ceRNA network was also analyzed. At the same time, two predictive nomograms were constructed, and their high AUC values suggested that they might be helpful for clinical oncologists to evaluate metastasis. Through the above analysis, the present study inferred that the metastasis-related ceRNAs of KPNB1, SGPL1, H2AFX, hsa-miR-30d-5p, hsa-miR-200c-3p and hsa-miR-130b-3p and metastasis-related immune cells of T cells CD4 memory resting and Macrophages M0 might play an important role in ACC metastasis.

Previous studies had shown that KPNB1 is associated with the occurrence of tumors [28]. Cell cycle regulation of KPNB1 suggested that its expression may be associated with proliferation, and KPNB1 showed comparatively high expression in tissues that proliferate actively [28]. KPNB1 proteins were the major nuclear receptor proteins in the cell, and proliferating cancer cells might regulate the expression of nuclear cytoplasmic transporter KPNB1 protein in varying degrees to maintain increased nuclear transporter [29]. Based on our analysis, we speculated that KPNB1 played an important role in the metastasis of adrenal cortical carcinoma. In addition, there might be some regulatory mechanism between hsa-mir-30d-5p and KPNB1, which made hsa-mir-30d-5p indirectly participate in the regulation of ACC metastasis. In fact, there was a regulatory mechanism between hsa-miR-30d and KPNB1 in malignant peripheral nerve sheath tumor (MPNST). In the present study, EZH2 enhanced the expression of the nuclear transport receptor KPNB1 by inhibiting hsa-miR-30d transcription via promoter binding activity [30].

Epithelial–mesenchymal transition (EMT) was a transcriptional process that played a key role in cancer metastasis [31]. Hsa-miR-200 family was a powerful EMT regulator by targeting ZEB1 and ZEB2, which regulated metastasis by regulating EMT [32]. Hsa-miR-200 regulated EMT and metastasis partially through a negative regulatory loop with the ZEB1/2 family of transcriptional suppressors [33]. Besides, hsa-miR-130b also regulated cell migration and invasion through EMT. It has been shown by RT-PCR and Western blotting that mir-130b is significantly up-regulated in BCA of bladder cancer, and mir-130b may be a potential target for BCA treatment. Hsa-miR-130b decreased the expression of IRF1, resulting in the repression of p-mTOR activity, and inhibited p-STAT3, p-AKT, p-ERK1/2 and EMT-related genes, eventually leading to the inhibition of cell migration and invasion [34]. However, there is no experimental evidence for the differential expression of mir-130b in acc. The present study provides a research direction for the future study of prognostic biomarkers of ACC.

Our study also suggested that H2AFX and SPGPL1 were associated with ACC metastasis. The human H2AX gene (H2AFX) maps to chromosome 11 at position 11q23, in a region that frequently exhibited mutations or deletions in a large number of human cancers [35]. H2AFX was a central component of numerous signaling pathways in response to DNA double-strand breaks (DSBs) [36]. The DSB was a serious lesion that can initiate genomic instability, ultimately leading to tumorigenesis [36]. Besides, SGPL1, a protein promoting cell apoptosis, was found to be expressed in the adrenal cortex, and down-regulated in tumor [37]. Thus, we speculated that H2AFX and SGPL1 might also play a role in the metastasis of ACC.

Next, we observed the differences in the components of immune cells between metastatic and non-metastatic ACCs, and found that T cells CD4 memory resting and Macrophages M0 may be related to ACC metastasis. The tumor microenvironment contains innate and adaptive immune cells, which display pro or anti-tumor functions [38]. Immunocytes, including T cell, NK cell and DCs, played a key role in immune responses of anti-tumors.

Macrophages were major players of tumor immunity [38]. Macrophages M0 can differentiate into Macrophages M1 and Macrophages M2 [39]. In general, Macrophages M1 were potent tumor-fighting cells, whereas Macrophages M2 displayed protumoral functions [38]. Mature macrophages were strategically distributed in the human body and perform important immune surveillance activities [39]. Macrophages M1 played a protective role in tumorigenesis, which activated tumor-killing mechanisms and antagonized the suppressive activities of TAMs, MDSCs, M2 macrophages, regulatory macrophages and immature myeloid cells, which had been proved to inhibit adaptive tumor-specific immune responses and promote tumor growth, invasion and metastasis [29,40]. By contrast, TAM isolated from solid and metastatic tumors showed inhibition of M2-like phenotype [39]. Furthermore, evidence accumulated from many cancer models suggested that macrophages contributed to the progression of tumors, and the increase of TAM, MDSC and immature monocytes was associated with poor prognosis [41,42]. Tumor associated macrophage TRMS can promote tumor growth by inhibiting antitumor immune response.

T cells can be divided into CD4 and CD8. Tumor growth was mainly controlled by CD4 and CD8 T cells [43]. Previous studies had found that T cells CD4+ memory were significantly correlated with the abundance of CCL5-related chemokine receptors [15]. CCL5 might induce the recruitment and activation of specific memory T cells by interacting with certain receptors on memory T cells [15]. CCR5 was the most famous receptor of CCL5 [15]. Previous studies had also shown that the CCL5–CCR5 axis played an active role in the tumorigenesis: as a growth factor, it stimulated angiogenesis and participated in the immune escape mechanism [44].

There were several limitations of our study that should be acknowledged. First, the amount of data released in publicly available datasets was limited, so the clinicopathological parameters analyzed in the present study were not comprehensive, which might lead to potential errors or deviations. Second, the heterogeneity of the immune micro-environment related to the location of immune infiltration was not considered. Third, the present study was only a correlation study on multiple dimensions rather than a biological mechanism study. However, based on the results of this correlation study, we will use biological experiments such as Luciferase reporter assay, Chromatin Immunoprecipitation (ChIP) to prove the direct interaction mechanism of ceRNAs in the future. Furthermore, we would like to demonstrate a molecular cross-talk between cancer cells and immune cells. For example, an exosome secreted by cancer cells contains ceRNAs, which work on immune cells to mediate the metastasis of adrenal carcinoma. Last but not least, the small sample size of adrenal cortical carcinoma may reduce the confidence and transformation of the predictive models into other cohorts. To reduce the bias causing by the small sample size, multiple databases were used to detect gene and protein expression levels of key biomarkers at the tissue and cell levels. The results showed the stability of the primary analysis results (Supplementary Figures S3–S10).

However, despite its limitations, the present study did first analyze the co-expression of ACC-specific tumor-infiltrating immune cells and ceRNA networks, construct the nomograms to predict the prognosis of ACC patients, and speculate that T cells CD4 memory resting, macrophages M0, KPNB1, SGPL1, H2AFX, hsa-miR-30d-5p, hsa-miR-200c-3p and hsa-miR-130b-3p might play an important role in ACC metastasis. The predictive nomograms proposed in the study might provide comprehensive clinical information for improving the personalized management of ACC patients. In the future, more data would be needed to improve the model. At present, there is no study on the direct molecular biological mechanism of metastatic ACC specific ceRNA and the intercellular communication between cancer cells and macrophage M0. Therefore, our study suggests that we can use the direct molecules of ACC specific ceRNA as the key molecules to study its influence on ACC transfer process and its mechanism. In combination with macrophage M0, the relationship and pathway between the key molecules and the metastasis of macrophage M0 in ACC were studied. As our future research direction, we would investigate the direct molecular biological mechanisms of metastatic ACC-specific ceRNAs and the intercellular communication between cancer cells and T cells CD4 memory resting and Macrophages M0.

## Conclusions

The present study constructed two nomograms based on tumor-infiltrating immune cells and ceRNA networks to predict metastasis of ACC patients, and demonstrated the utility of their high AUC values. The predictive models proposed in the study may provide much-needed comprehensive clinical information for improving the personalized management of ACC patients. Moreover, the present study inferred that KPNB1, SGPL1, H2AFX, hsa-miR-30d-5p, hsa-miR-200c-3p, hsa-miR-130b-3p, T cells CD4 memory resting and Macrophages M0 might play an important role in ACC metastasis.

## Supplementary Material

Supplementary Figures S1-S10 and Tables S1-S2

## Data Availability

All datasets for this study are included in the TCGA-ACC program.

## Abbreviations

ACC: adrenal cortical carcinoma
AUC: area under curve
ceRNA: competitive endogenous RNA
ChIP: chromatin immunoprecipitation
CIBERSORT: cell type identification by estimating relative subsets of RNA transcripts
DSB: double-strand break
EMT: epithelial–mesenchymal transition
FDR: false discovery rate
MRE: microRNA response element
ROC: receiver operating characteristic curves
SD: standard deviation
TCGA: The Cancer Genome Atlas
