# Supplementary material for: The construction and analysis of tumor-infiltrating immune cells and ceRNA networks in metastatic adrenal cortical carcinoma
Source: Biosci Rep. 2020 Mar 27;40(3):BSR20200049. doi: 10.1042/BSR20200049 (PMC7103591; doi:10.1042/BSR20200049)
Supplement: Supplementary Figures S1-S10 and Tables S1-S2 [file BSR-2020-0049_supp.pdf]

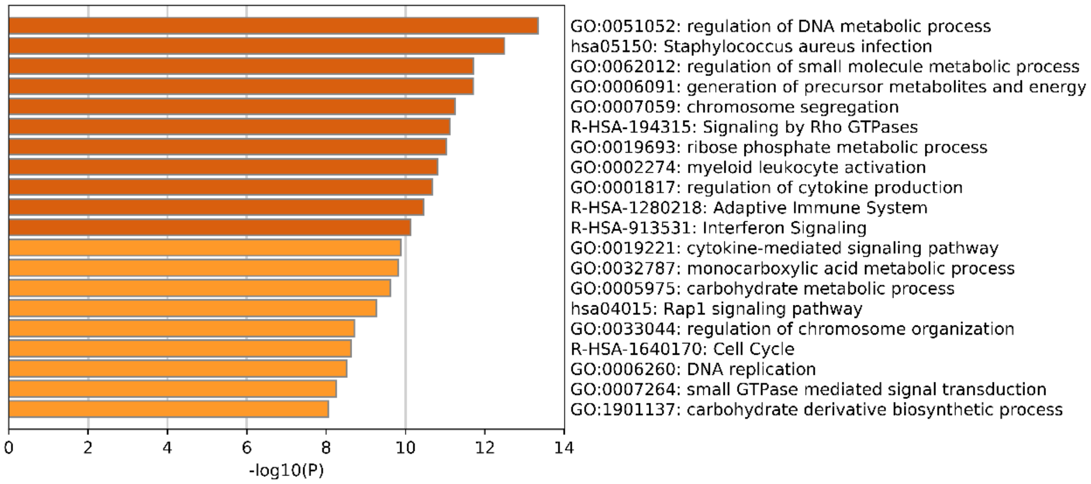

Figure S1 The results of the enrichment analysis of differentially expressed genes associated with metastasis, which revealed immune system and genetic material biological processes or pathways.

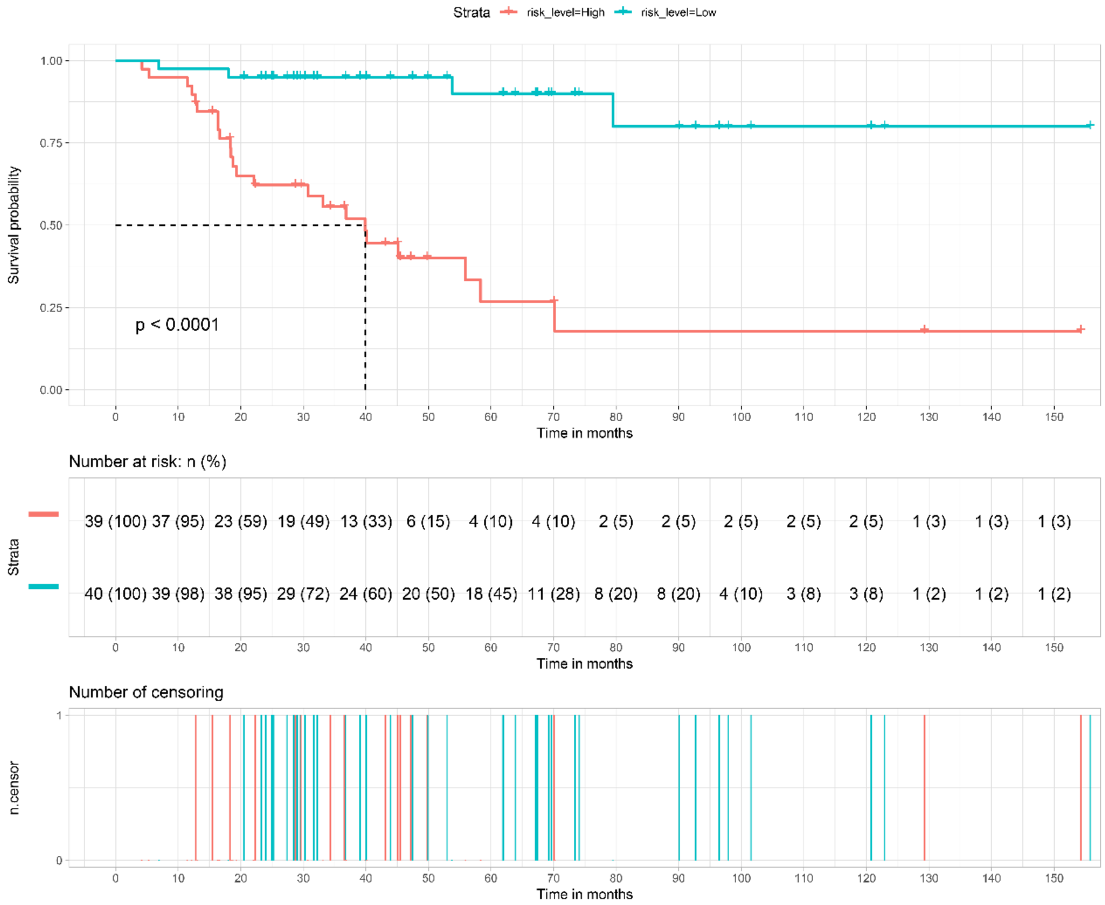

Figure S2 The Kaplan–Meier survival curve revealed a significant correlation between risk level defined by the multivariable model including immune cells and survival of ACC (P < 0.001).

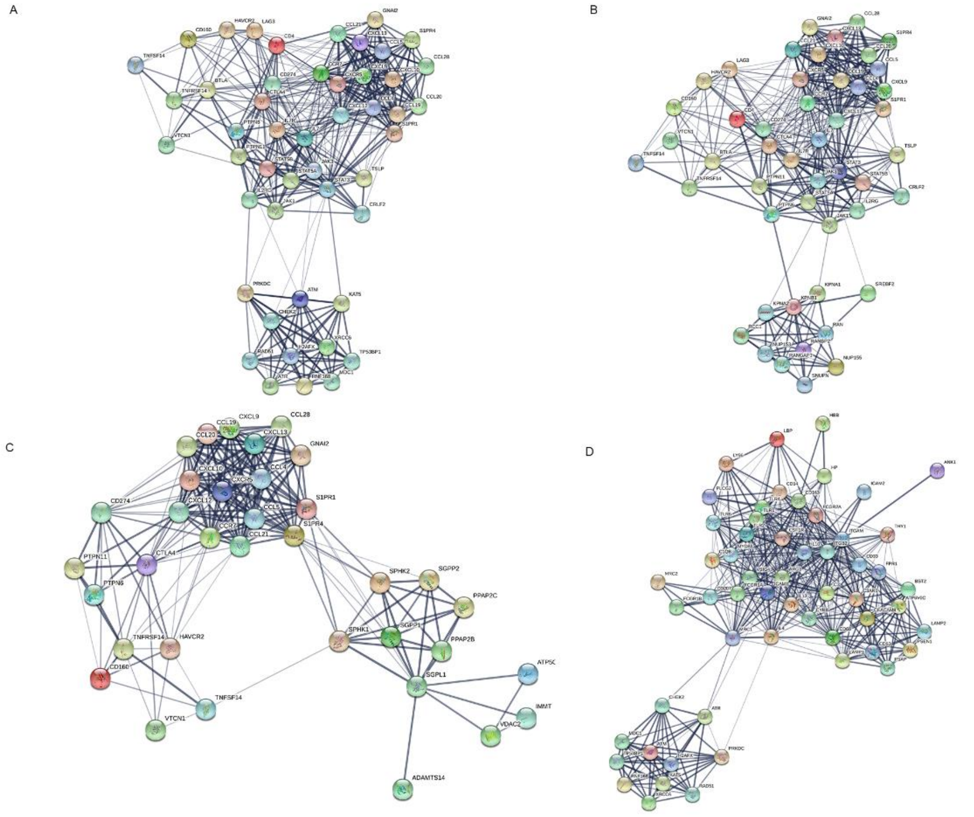

Figure S3 Protein-protein interaction network of ceRNAs and corresponding cell markers.

T cells cell marker and H2AFX (A), T cells and KPNB1 (B), T cells cell marker and SGPL1 (C), and macrophages cell marker and H2AFX (D).

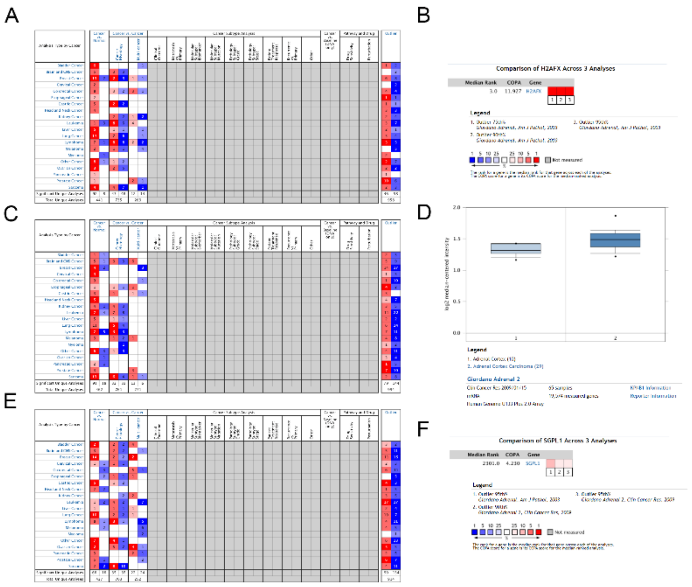

Figure S4 In Oncomine database, KPNB1 (P < 0.001) (C, D) were significantly differentially expressed between ACC and normal adrenal tissue, H2AFX (Median rank 3.0, COPA = 11.927) (A, B) and SGPL1 (Median rank 2301.0, COPA = 4.230) (E, F) were abnormal upregulated in ACC in the outlier analysis across multiple studies.

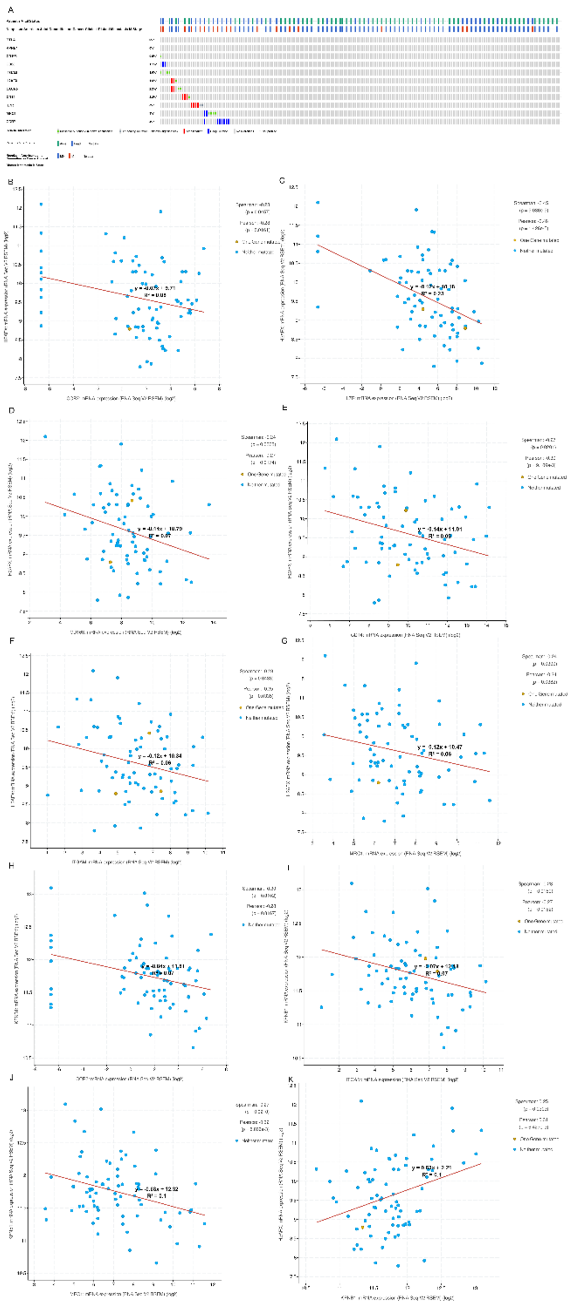

Figure S5 The results of genomics and clinical data analyzed by cBioPortal. Comprehensive analysis of genomics and clinical data in cBioPortal database showed that CCR7, CXCR5, IL7R CD68, CD163, CD14, ITGAM, MRC1 and H2AFX had genomic alteration in primary ACC (A). In addition, H2AFX had significant co-expression patterns with markers of T cells CD4 memory CCR7 (P = 0.046), IL7R (P < 0.001) and macrophage M0 markers CD163 (P = 0.037), CD14 (P = 0.020), ITGAM (P = 0.043) and MRC1 (P = 0.038) in ACC (B-G). KPNB1 also had significant co-expression with CCR7 (P = 0.010), ITGAM (P = 0.016), MRC1(P = 0.021) and H2AFX (P = 0.006) (H - K).

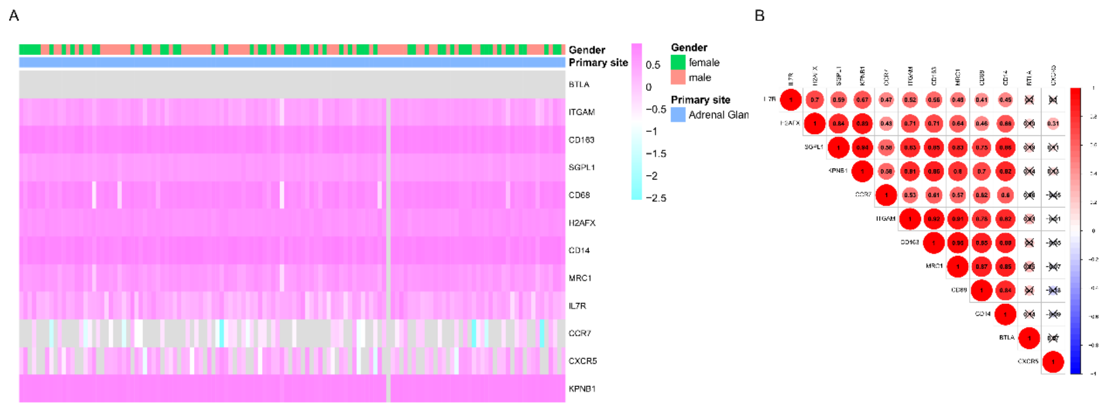

Figure S6 The heatmap (A) and co-expression analysis (B) of KPNB1, H2AFX, SGPL1 and cell markers.

In the normal adrenal tissues in the GTEx database, KPNB1, H2AFX, SGPL1 had significant co-expression patterns with almost all cell markers (CCR7, IL7R CD68, CD163, CD14, ITGAM, MRC1).

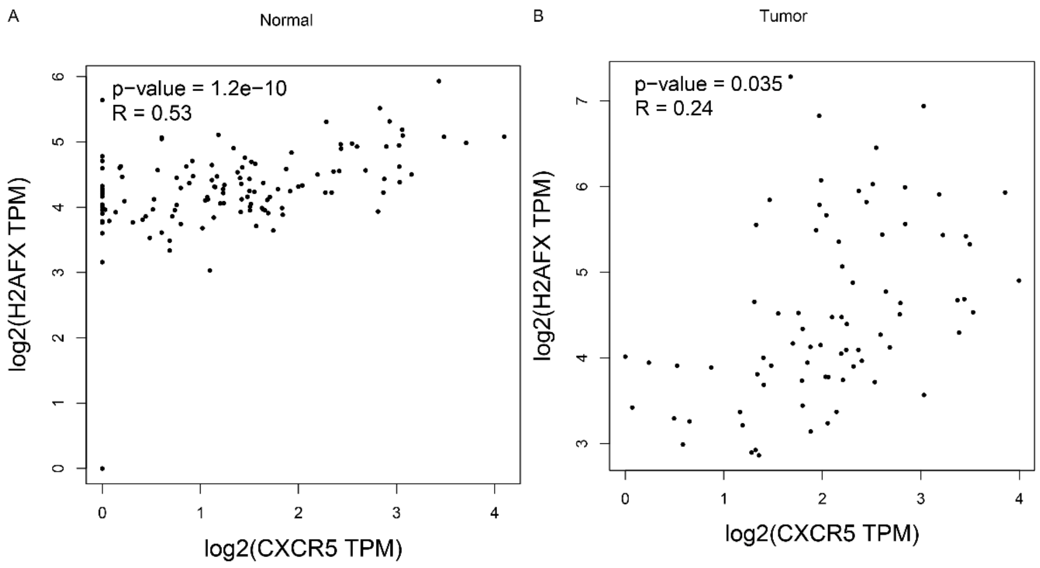

Figure S7 In GEPIA database, H2AFX and CXCR5 were co-expressed in normal adrenal tissues (A) and ACC (B).

GEPIA database analysis showed the co-expression pattern of H2AFX and CXCR5 in normal adrenal tissues and ACC.

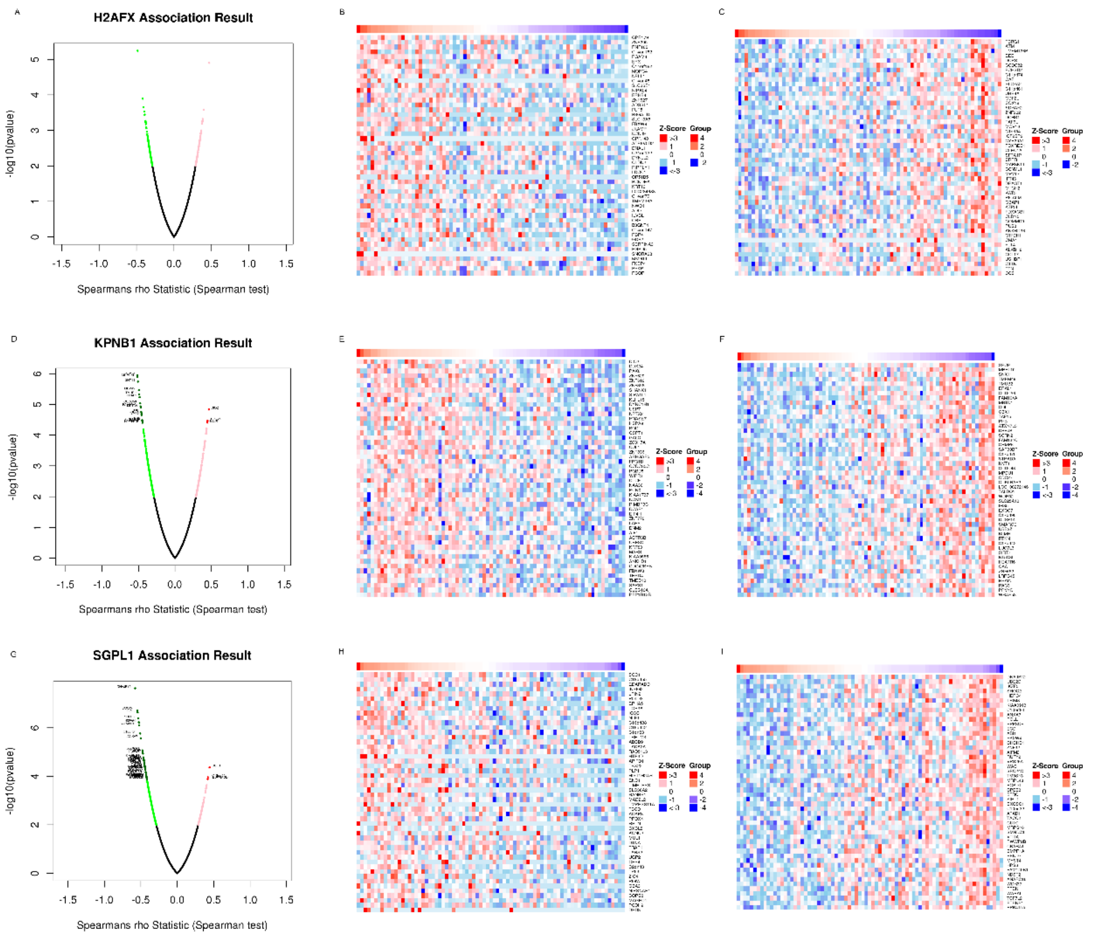

Figure S8 The data mining results of LinkedOmics database.

The LinkedOmics database showed that KPNB1 (A, B, C), H2AFX (D, E, F) and SGPL1 (G, H, I) had significant co-expression of many immune-related proteins in ACC.

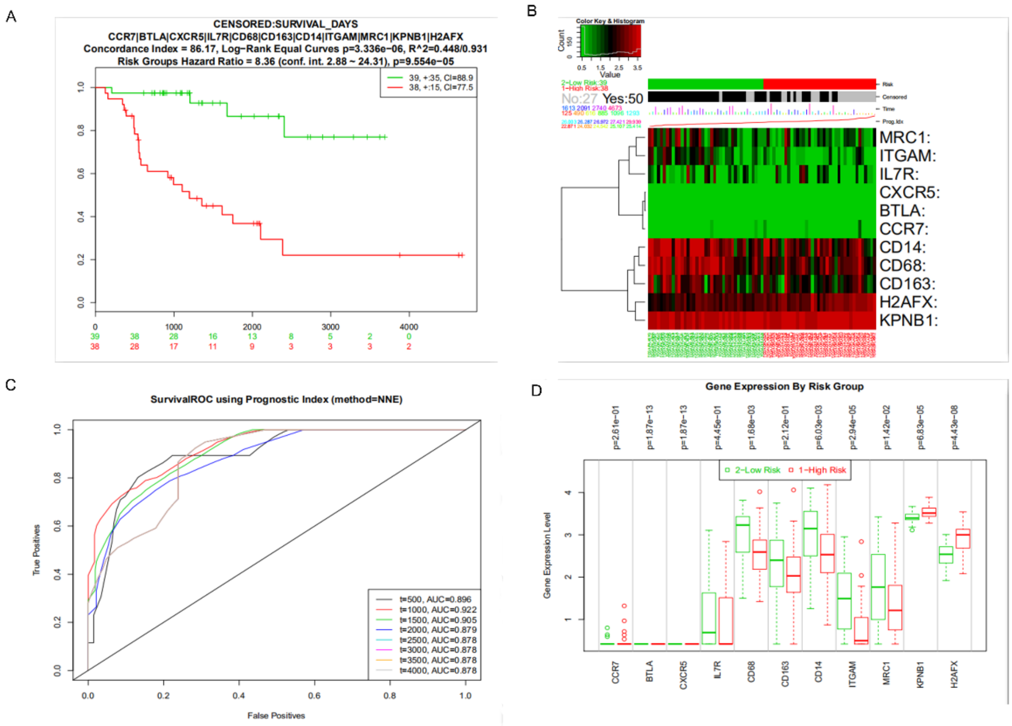

Figure S9 The analysis results of SurvExpress database for external validation.

The analysis results of SurvExpress database suggested that these genes had significant predictive value for prognosis (Censoring event: overall death, Hazard Ratio = 8.36 (95% CI, 2.88 to 24.31), P < 0.001) in external validation (A, B). The ROC curve showed decent predictive accuracy of the model (C) and the boxplot illustrated that CXCR5, CD68, CD14, ITGAM, MRC1 and H2AFX were differentially expressed between normal adrenal tissues and ACC (D).

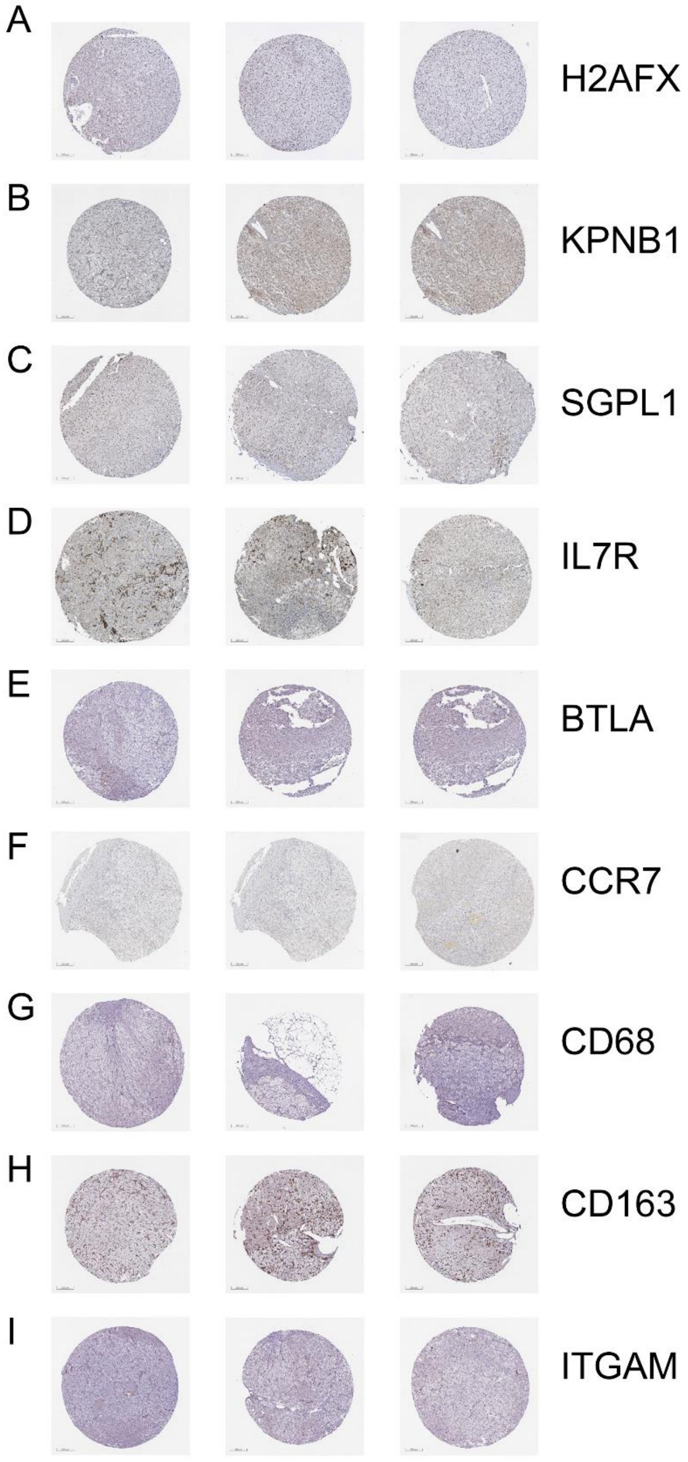

Figure S10 The Human Protein Atlas data mining results.

The Human Protein Atlas data mining results showed that proteins of H2AFX (A), SGPL1 (C), IL7R (D), BTLA (E), CCR7 (F), CD68 (G), CD163 (H), ITGAM (I) could not be detected in normal tissues, and KPNB1 (B) had low expression in normal adrenal tissues.

Table S1 Baseline information of 92 patients diagnosed with adrenal cortical carcinoma available from the TCGA

Variables	Total Patients (N = 92)
**Age, years**	
Mean ± SD	47.16 ± 16.21
Median (Range)	48.50 (14 - 83)
**Gender**	
Female	60 (65.22%)
Male	32 (34.78%)
**Race**	
Asian	2 (2.17%)
Black or African American	1 (1.09%)
White	78 (84.78%)
Unknown	11 (11.96%)
**Clinical M stage**	
M1	18 (19.57%)
M0	72 (78.26%)
Unknown	2 (2.17%)

Abbreviations: SD, Standard deviation.

Table S2 The analysis results of OncomiR database

miRNA Name	Cancer Abbreviation	Clinical Parameter	ANOVA P-value
hsa-miR-30d-5p	BLCA	Histologic Grade	1.11E-08
hsa-miR-30d-5p	KIRC	Histologic Grade	5.23E-07
hsa-miR-30d-5p	KICH	Pathologic T Status	2.76E-06
hsa-miR-30d-5p	KIRC	Pathologic T Status	4.21E-06
hsa-miR-30d-5p	STAD	Histologic Grade	2.59E-05
hsa-miR-30d-5p	BLCA	Pathologic Stage	1.23E-04
hsa-miR-30d-5p	TGCT	Clinical T Status	1.99E-04
hsa-miR-30d-5p	STAD	Pathologic T Status	4.80E-04
hsa-miR-30d-5p	ESCA	Pathologic T Status	5.71E-04
hsa-miR-30d-5p	BLCA	Pathologic T Status	7.67E-04
hsa-miR-30d-5p	STAD	Pathologic Stage	8.42E-04
hsa-miR-30d-5p	BRCA	Pathologic N Status	2.63E-03
hsa-miR-30d-5p	PAAD	Histologic Grade	2.96E-03
hsa-miR-30d-5p	LIHC	Histologic Grade	4.29E-03
hsa-miR-30d-5p	TGCT	Pathologic T Status	4.37E-03
hsa-miR-30d-5p	BLCA	Pathologic M Status	1.71E-02
hsa-miR-30d-5p	HNSC	Pathologic T Status	1.73E-02
hsa-miR-30d-5p	LUAD	Pathologic T Status	1.85E-02

hsa-miR-30d-5p	ACC	Pathologic T Status	2.23E-02
hsa-miR-30d-5p	KIRC	Pathologic M Status	2.28E-02
hsa-miR-30d-5p	TGCT	Pathologic M Status	2.33E-02
hsa-miR-30d-5p	TGCT	Clinical M Status	2.42E-02
hsa-miR-30d-5p	LUSC	Pathologic T Status	2.84E-02
hsa-miR-30d-5p	HNSC	Clinical T Status	2.96E-02
hsa-miR-30d-5p	KICH	Pathologic M Status	3.08E-02
hsa-miR-30d-5p	THCA	Pathologic T Status	3.66E-02
hsa-miR-30d-5p	COAD	Pathologic Stage	3.69E-02
hsa-miR-30d-5p	LIHC	Pathologic Stage	4.20E-02
hsa-miR-30d-5p	LUSC	Sex	4.34E-02
hsa-miR-30d-5p	ACC	Clinical M Status	4.43E-02
hsa-miR-30d-5p	LUAD	Pathologic N Status	4.73E-02
hsa-miR-200c-3p	KIRC	Histologic Grade	4.50E-08
hsa-miR-200c-3p	OV	Histologic Grade	4.98E-08
hsa-miR-200c-3p	STAD	Histologic Grade	1.57E-06
hsa-miR-200c-3p	BLCA	Pathologic M Status	4.84E-06
hsa-miR-200c-3p	LGG	Histologic Grade	5.89E-06
hsa-miR-200c-3p	KIRP	Clinical N Status	7.67E-06
hsa-miR-200c-3p	PAAD	Histologic Grade	1.09E-05

hsa-miR-200c-3p	KIRP	Pathologic N Status	1.37E-05
hsa-miR-200c-3p	BLCA	Histologic Grade	9.69E-05
hsa-miR-200c-3p	BLCA	Pathologic Stage	1.12E-03
hsa-miR-200c-3p	BLCA	Pathologic T Status	1.19E-03
hsa-miR-200c-3p	ACC	Clinical M Status	1.32E-03
hsa-miR-200c-3p	KIRP	Pathologic M Status	1.61E-03
hsa-miR-200c-3p	COAD	Pathologic M Status	1.99E-03
hsa-miR-200c-3p	STAD	Pathologic T Status	2.24E-03
hsa-miR-200c-3p	OV	Clinical Stage	2.53E-03
hsa-miR-200c-3p	UCEC	Histologic Grade	2.80E-03
hsa-miR-200c-3p	READ	Pathologic M Status	4.95E-03
hsa-miR-200c-3p	KICH	Pathologic T Status	6.39E-03
hsa-miR-200c-3p	LUSC	Sex	7.69E-03
hsa-miR-200c-3p	STAD	Pathologic N Status	8.12E-03
hsa-miR-200c-3p	STAD	Pathologic Stage	9.46E-03
hsa-miR-200c-3p	LIHC	Pathologic T Status	9.60E-03
hsa-miR-200c-3p	PAAD	Pathologic M Status	1.02E-02
hsa-miR-200c-3p	LIHC	Histologic Grade	1.03E-02
hsa-miR-200c-3p	LIHC	Pathologic Stage	1.45E-02
hsa-miR-200c-3p	ACC	Pathologic Stage	1.54E-02

hsa-miR-200c-3p	TGCT	Pathologic M Status	1.96E-02
hsa-miR-200c-3p	TGCT	Pathologic Stage	2.00E-02
hsa-miR-200c-3p	BRCA	Pathologic T Status	2.07E-02
hsa-miR-200c-3p	KIRP	Clinical T Status	2.24E-02
hsa-miR-200c-3p	UCS	Clinical Stage	2.53E-02
hsa-miR-200c-3p	KIRP	Sex	2.86E-02
hsa-miR-200c-3p	LIHC	Sex	2.87E-02
hsa-miR-200c-3p	TGCT	Clinical Stage	2.96E-02
hsa-miR-200c-3p	UCEC	Clinical Stage	3.49E-02
hsa-miR-200c-3p	LUAD	Sex	3.92E-02
hsa-miR-200c-3p	CHOL	Histologic Grade	3.96E-02
hsa-miR-200c-3p	KIRP	Pathologic Stage	4.30E-02
hsa-miR-200c-3p	TGCT	Pathologic N Status	4.82E-02

Abbreviations: ACC: adrenocortical carcinoma; BLCA: bladder urothelial carcinoma; BRCA: breast invasive carcinoma; CHOL: cholangiocarcinoma; COAD: colon adenocarcinoma; KICH: kidney chromophobe; KIRC: kidney renal clear cell carcinoma; KIRP: kidney renal papillary cell carcinoma; LGG: brain lower grade glioma; LIHC: liver hepatocellular carcinoma; LUAD: lung adenocarcinoma; LUSC: lung squamous cell carcinoma; OV: ovarian serous cystadenocarcinoma; PAAD: pancreatic adenocarcinoma; READ: rectal adenocarcinoma; STAD: stomach adenocarcinoma; TGCT: testicular germ cell tumors; UCEC: uterine corpus endometrial carcinoma; UCS: uterine carcinosarcoma
